# Regulation of Cellulase and Hemicellulase Gene Expression in Fungi

**DOI:** 10.2174/1389202911314040002

**Published:** 2013-06

**Authors:** Antonella Amore, Simona Giacobbe, Vincenza Faraco

**Affiliations:** 1 Department of Chemical Sciences, University of Naples “Federico II”, Complesso Universitario Monte S. Angelo, via Cintia, 4 80126 Naples, Italy;; 2 School of Biotechnological Sciences, University of Naples “Federico II” Italy

**Keywords:** Cellulase, Cellulose, CRE1, Hemicellulase, Sophorose, Xylan, XYR1.

## Abstract

Research on regulation of cellulases and hemicellulases gene expression may be very useful for increasing the production of these enzymes in their native producers. Mechanisms of gene regulation of cellulase and hemicellulase expression in filamentous fungi have been studied, mainly in *Aspergillus* and *Trichoderma*. The production of these extracellular enzymes is an energy-consuming process, so the enzymes are produced only under conditions in which the fungus needs to use plant polymers as an energy and carbon source. Moreover, production of many of these enzymes is coordinately regulated, and induced in the presence of the substrate polymers. In addition to induction by mono- and oligo-saccharides, genes encoding hydrolytic enzymes involved in plant cell wall deconstruction in filamentous fungi can be repressed during growth in the presence of easily metabolizable carbon sources, such as glucose. Carbon catabolite repression is an important mechanism to repress the production of plant cell wall degrading enzymes during growth on preferred carbon sources. This manuscript reviews the recent advancements in elucidation of molecular mechanisms responsible for regulation of expression of cellulase and hemicellulase genes in fungi.

## 1 INTRODUCTION

In order to enhance energy security and mitigate climate change, interest in finding renewable fuels to replace petroleum-based ones is enormously increasing. The biofuels ethanol and biodiesel represent potential options for meeting these needs in the transportation sector. The uniqueness of cellulosic ethanol as a sustainable liquid transportation fuel, which can be produced in high volumes and at low cost, and its many powerful benefits have been recognized for decades [1-5]. A recent awareness of the urgent need to advance cellulosic ethanol production is evidenced by the number of reviews reported on the theme of ethanol fuel production from lignocellulosic biomass, with great attention to ethanol production from lignocellulosic residues, such as crop and wood residues and municipal solid waste [6-15]. The key step for conversion of lignocellulosic biomass into fermentable sugars for fuel production is represented by the hydrolysis of polysaccharides, resulting from biomass pretreatment, by cellulases and hemicellulases. Filamentous fungi are the major source of cellulases and hemicellulases. As far as cellulases are concerned, three main enzymatic activities are involved in cellulose hydrolysis: 1) endoglucanases (EC 3.2.1.4); 2) exoglucanases, including d-cellodextrinases (EC 3.2.1.74) and cellobiohydrolases (EC 3.2.1.91); and 3) β-glucosidases (EC 3.2.1.21). As far as hemicellulases are concerned, endo-β-1,4-xylanases (EC 3.2.1.8) and β-xylosidases (EC 3.2.1.37) are required for degradation of the xylan backbone, while auxiliary enzymes such as α-glucuronidases (EC 3.2.1), α-arabinofuranosidases (EC 3.2.1.55), acetylesterases or acetyl xylan esterases (EC 3.1.1.6) are required to achieve the complete degradation of complex substituted xylans. Research on regulation of cellulase and hemicellulase genes’ expression may be very useful for increasing production of these enzymes in their native producers. Mechanisms of cellulase and hemicellulase genes regulation have been studied in filamentous fungi, mainly in *Aspergillus* [16, 17] and *Trichoderma *[18]. The production of these extracellular enzymes is an energy-consuming process, so the enzymes are produced only under conditions in which the fungus needs to use plant polymers as an energy and carbon source. Moreover, production of many of these enzymes is coordinately regulated, and induced in the presence of the substrate polymers. Induction mechanisms of cellulase and hemicellulase genes expression involve activation of gene expression by the respective hydrolysis and/or transglycosylation products of cellulose and/or xylan, such as gentiobiose for *Penicillium* [19], and sophorose for *A. terreus* and *T. reesei *[20, 21]. In addition to induction by mono- and oligo-saccharides, genes encoding hydrolytic enzymes involved in plant cell wall deconstruction in filamentous fungi can be repressed during growth in the presence of easily metabolizable carbon sources, such as glucose. Carbon catabolite repression (CCR) is an important mechanism to repress the production of plant cell wall degrading enzymes during growth on preferred carbon sources [22-25].

This manuscript reviews the recent advancements in elucidation of molecular mechanisms responsible for regulation of expression of cellulase and hemicellulase genes in fungi.

## 2 REGULATION OF PRODUCTION OF CELLULASES AND HEMICELLULASES IN *TRICHODERMA REESEI*

The cellulolytic machinery of *T. reesei* is one of the most widely studied [26]. *T. reesei* genome *(http://genome.jgi psf.org/Trire2/Trire2.home.html*) contains ten cellulase and sixteen hemicellulase genes [27]. The enzymes so far identified and characterized as responsible for the cellulolytic activity of *T. reesei *include five endoglucanases -EGI/Cel7B, EGII/Cel5A, EGIII/Cel12A [28, 29], EGIV/Cel61A [30], and EGV/Cel45A [31] and two exoglucanases -the cellobiohydrolases CBHI/Cel7A and CBHII/Cel6A [32]. These enzymes act synergistically to convert cellulose into cellobiose [28-33], whose hydrolysis into glucose involves then two β-glucosidases -BGLI/Cel3A [34] and BGLII/Cel1A [35]. An additional protein, swollenin (encoded by the gene *swo1*), has been described, that disrupts crystalline cellulose structures, presumably making polysaccharides more accessible to hydrolysis [36]. The cellulases CBHI/Cel7A, CBHII/ Cel6A, EGI/Cel7B, and EGII/Cel5A are the most abundantly produced by *T. reesei* secreting them up to 40 g/liter [37]. Due to the enormous level of cellulase production, *T. reesei *revealed to be a potential candidate for advancing cellulosic ethanol by I Consolidated BioProcessing, engineering it to ferment monosaccharides into ethanol in high yields [38].

The *T. reesei* genome also contains sixteen hemicellulases including two GH43, one GH10, four GH11, one GH74, one GH62, two GH54, one GH67 and four GH95 [27]. Among these, two major endo-β-1,4-xylanases XYNI and XYNII (EC 3.2.1.8) [39]; and one β-xylosidase, BXLI (EC 3.2.1.37) [40] have been characterized.

The presence of cellulose, xylan or mixtures of plant polymers in the fungal culture medium causes abundant production of cellulolytic and xylanolytic activities by *T. reesei*, as already reported by the earlier studies [41-44]. Pure (oligo)saccharides, such as sophorose [20, 21], β-cellobiono-1,5-lactone, D-xylose, xylobiose, galactose, and lactose, have been also reported to induce cellulase and hemicellulase production in *T. reesei *(Table **1**) [24, 45-49].

Inability of the fungal cells to incorporate insoluble polymeric compounds, such as cellulose and xylan, aroused the question on how these polymers can initiate production of hydrolytic enzymes. Several studies investigating this aspect postulated the inducer function of a low molecular weight and soluble compound derived from cellulose. One of the proposed mechanisms is that the fungus produces basal levels of cellulase (mainly CEL7A and CEL6A) and that the activity of these extracellular enzymes on cellulose produces a soluble inducer, which can enter the cell and affect induction [50, 51]. In support of this mechanism, it was shown that antibodies against CBHI, CBHII, EGI and EGII blocked the expression of *cbh1/cel7a *gene in the presence of cellulose but not the soluble inducer sophorose [50]. The constitutive levels of these cellulases and their role in cellulase induction were afterwards demonstrated by Carle-Urioste *et al.* [51]. These authors showed that the mRNAs *cbh1* and *egl1* are transcribed under uninduced conditions, and that induction with cellulose results in at least 1100-fold increase of both transcripts, as demonstrated by Northern blots. The basal activity of the *cbh1* promoter was also examined by using a chimeric vector in which the gene encoding hygromycin B phosphotransferase [52] was placed under the control of the 59-flanking DNA sequence of the *cbh1* gene. Under uninduced conditions, resistance to the antibiotic hygromycin B was observed with *T. reesei* cells transformed with this vector and grown on medium lacking cellulose. An antisense RNA strategy was also adopted by the same authors to gain *in vivo* evidence for the requirement of the basal expression of the cellulase in induction of the cellulase transcripts by cellulose [51]. The results demonstrated that the expression of this antisense RNA produced marked effects on the induction of the *cbh1* transcript using cellulose (reduction of the *cbh1* transcript expression between 80 and 90%) but not sophorose as an inducer. The authors also showed that the initial hydrolysis of cellulose is the rate-limiting step in the induction, as suggested by the observation that the addition of the cellulase system or its purified enzyme members to a culture of *T. reesei*, in the presence of cellulose, resulted in earlier detection of the *cbh1* and *egl1* transcripts. The time required for induction of *cbh1* and *egl1* transcripts using cellulose, cellulose + cellulase, or sophorose is 14, 10, and 4 h, respectively. This result supports the hypothesis that oligosaccharide( s) is(are) formed *in vivo* from cellulose by the activity of a low, constitutive, and extracellular cellulase activity. The relatively slow induction by sophorose could be explained by the fact that the inductive process is protein synthesis-dependent. In addition, it has recently been shown that a sophorose-inducible β-diglucoside permease is involved in the induction of the cellulase system in *T. reesei* [53]. Subsequently, Foreman *et al.* [54] identified further genes whose regulatory behavior is consistent with their role in primary inducer formation for cellulase expression. Among them, the mRNA of *cel5b *was moderately expressed during growth on glycerol, glucose, sophorose and lactose, and only slightly induced over this level by cellulose. It is worth noting that CEL5B contains the *consensus* sequence for membrane-anchoring via a glycosylphosphatidylinositol residue. All these properties make it an interesting candidate for generating the inducer of cellulase formation. Similarly, the acetyl xylan esterase Axe2, which is also predicted to contain a glycosylphosphatidylinositol anchor, may be involved in primary induction of some hemicellulases [54].

The surface-bound cellulolytic activity displayed by conidia of *T. reesei, *mainly due to CEL6A/CBHII [55, 56] is also considered important for cellulase induction since its elimination by detergents hinders germination of the conidia on cellulose. These conclusions were deduced by the observation that introduction of multiple copies of the *cel6a *gene into *T. reesei *caused an enhanced secretion of CEL7A and CEL6A on cellulose and an increased cellulase activity on cellulose corresponding to enhanced level of conidial-bound CEL6A [56, 57]. Consistently, a *cel6a *knocked out strain showed a delay in growth and cellulase formation on cellulose [58]. In more details, comparing strains in which the corresponding genes of the main cellulases (*cel6a, cel7a, cel7b, cel5a*) had been deleted, Seiboth *et al.* [58] showed that strains knocked out for *cel6 *and *cel5a*, respectively, exhibited a significantly reduced expression of the remaining cellulase genes, while strains carrying the *cel7a *or *cel7b *deletion showed these transcripts. A strain showing both the cellobiohydrolases *cel6a *and *cel7a *deletion, was unable to initiate growth on cellulose. During growth on lactose, these strains showed no significant alteration in their ability to express the respective other cellulase genes. These data support the role of CEL6A and other conidial-bound cellulases (such as CEL5A, for which a conidial location is not yet known) in the induction of cellulases and germination on cellulose. 

Ilmèn *et al.* [59] investigated basic features of expression regulation of the *T. reesei* cellobiohydrolases *cbh1 *and *cbh2 *and endoglucanases* egl1*, *egl2 *and *egl5 *encoding genes, at the mRNA level, showing that these cellulase genes are coordinately expressed and the steady-state mRNA levels of *cbh1*/Cel7A is the highest. The highest induction level was achieved with cellulose and sophorose and moderate expression was observed when cellobiose or lactose were used as the carbon source. No expression could be observed on glucose-containing medium and high glucose levels abolish the inducing effect of sophorose. However, derepression of cellulase expression occurs without apparent addition of an inducer once glucose has been depleted from the medium. This expression seems not to arise simply from starvation, since the lack of carbon or nitrogen as such is not sufficient to trigger significant expression. It was also found that glycerol and sorbitol do not promote expression but, unlike glucose, do not inhibit it either, because the addition of 1 to 2 mM sophorose to glycerol or sorbitol cultures provokes high cellulase expression levels. 

The best inducer of cellulase expression so far known is sophorose ((2-*O*-*β*-glucopyranosyl-D-glucos) [60, 21, 61], whose synthesis from cellobiose involves the transglycosylation activity of *β*-glucosidase [62]. Induction by sophorose is affected by various parameters such as its concentration and rate of uptake [61, 63]. Two pathways of sophorose utilisation were for the first time hypothesised by Loewenberg and Chapman [64]: a catabolic pathway characterized by a high capacity but low affinity for sophorose, and a cellulase inducing pathway endowed with a lower capacity but higher affinity for sophorose. As a matter of fact, Kubicek *et al.* [53] showed that sophorose is transported by a cellobiose permease, characterized by low K_M_ and V_max_ for sophorose, and thus competing with the extracellular β-glucosidase, which has a much higher K_M_ but also V_max_ for it. 

Most authors implied a β-glucosidase in the process of sophorose production. *T. reesei *produces β-glucosidases having different cellular localizations [65-69]. The gene *cel3a *[65, 70] encodes the major extracellular β-glucosidase identified as one of the β-glucosidases involved in inducer formation. Knock-out of the *cel3a *gene causes a delay in induction of the other cellulase genes by cellulose, but not by sophorose, whilst a *cel3a*-multicopy strain is able to produce higher levels of cellulases than the wild-type strain under nonsaturating concentrations of sophorose, but both strains were comparably efficient at saturating concentrations [48]. The observation that the β-glucosidase inhibitor nojirimycin inhibits cellulase induction also in the *cel3a *disrupted strain suggests that the CEL3A is not the only β-glucosidase involved in inducer formation [48]. An additional β-glucosidase-encoding gene has been cloned [35] and properties and intracellular localisation of the corresponding enzyme have been characterised [69]. However, as no multicopy or gene deletion studies have yet been carried out, ascertainment of its involvement in cellulase induction requires further investigation.

### 2.1 Transcriptional Factors Involved in Regulation of Cellulase and Hemicellulase Genes’ Expression in *T. reesei*

**Fig. (1):**
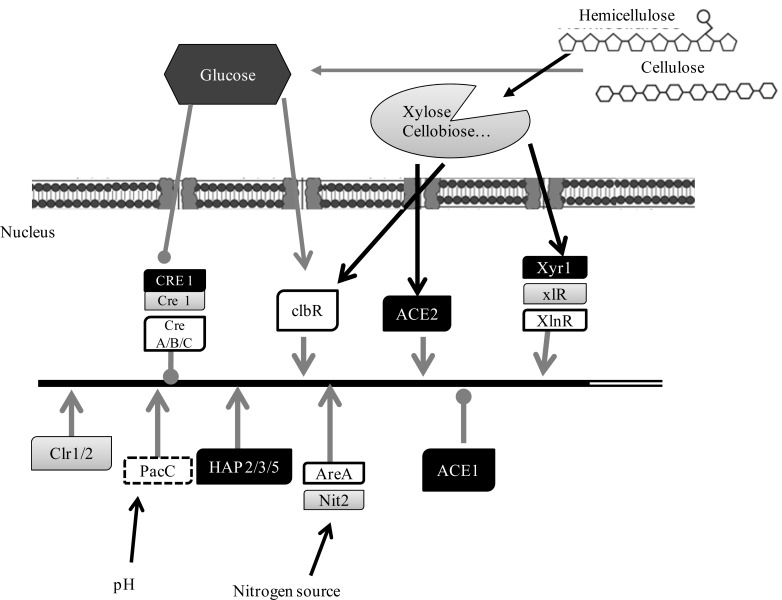
Schematic representation of transcriptional factors affecting cellulases and xylanases expression in *T. reseei* (black box), *N. crassa* (grey box) and *Aspergillus* spp. (white box). The carbon catabolite repressor CRE, the activators clbR, Xyr/xlR/XlnR,Clr,ACE2, the repressor ACE1, the CCAAT binding Hap2/3/5 complex, the pH regulator PacC, and the nitrogen regulators AreA and Nit2 are shown. The repression activity (
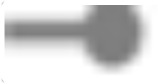
), the induction activity (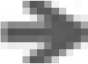) and also promoter (
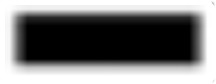
) and coding region (
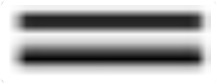
) are indicated.

Foreman *et al.* [54] performed investigations on regulation of cellulase and hemicellulase genes’ expression in *T. reesei* by microarrays showing that most of the genes encoding known and putative biomass-degrading enzymes are transcriptionally co-regulated. This co-regulation indicates a tightly coordinated cooperation of the corresponding transcription factors, five of which have been so far identified (Fig. **1**): the positive regulators XYR1, ACE2 and the HAP2/3/5 complex, the repressor ACE1 and the carbon catabolite repressor CRE1 (Table **2**) [25]. 

The main positive regulator of cellulase and hemicellulase gene expression is represented by XYR1 (xylanase regulator 1) [48, 18], a zinc binuclear cluster protein binding to a GGCTAA-motif arranged as an inverted repeat [48]. *xyr1 *deletion abolishes cellulase induction on cellulose and sophorose and impairs the induction of hemicellulase genes involved in xylan and arabinan degradation [71, 18], thus proving its essential role in the induction process. *xyr1 *transcription seems not to be induced during growth on cellulose [72]. Most of eukaryotic transcriptional activators are present in cells only in small amounts required to start gene expression [73], and, in many cases, they are further induced by the conditions for which they are needed and are degraded once they are no longer required [74]. On the contrary, *xyr1 *expression is regulated solely by CRE1-dependent CCR and by repression by the specific transcription factor ACE1, not by induction [72, 49]. Whether an increase in constitutive expression of *xyr1 *would increase enzyme formation is not sufficiently understood. Aigner-Mach *et al.* [72] fused the *xyr1 *gene under the regulatory signals of the *nag1 *(N-acetyl-β-D-glucosaminidase) promoter, which resulted in a slightly earlier beginning of xylanase formation but did not significantly enhance the final enzyme titre. However, these studies used the uninduced, basal expression level of *nag1*, which is not much higher than that of *xyr1 *itself, and studies using stronger expressed promoters (such as those for glycolytic or hydrophobin genes) must be used to clarify whether the constitutive expression of *xyr1 *would enhance cellulase and/or xylanase formation.

The cellulase activator ACE2 also belongs to the class of zinc binuclear cluster proteins [75]. It has so far been shown to occur only in *Trichoderma *spp. Deletion of *ace2 *lowers the transcript levels of the major cellulases and causes a decrease of cellulase activity during growth on cellulose [75, 76], whilst it does not affect cellulase induction by sophorose [75]. It is worth noting that the DNA-binding domain of ACE2 is able to bind to the promoter motif [GGC(T/A)4] present in the *cbh1 *promoter also recognized by XYR1 [77]. Stricker *et al.* [76] suggested that phosphorylation and dimerization are needed for the binding of ACE2 to the corresponding promoter element. 

The CCAAT motif is a common *cis*-acting element found in either orientation in the promoter and enhancer region of a large number of eukaryotic genes. Particularly, in yeasts, as well in filamentous fungi, the CCAAT box-binding proteins identified so far all belong to the group of HAP-like factors. Site-directed mutagenesis of the promoter of one of the most abundant cellulase produced by *T. reesei*, *cbh2*, revealed the existence of an undecameric nucleotide motif which is essential for gene expression *in vivo*. Moreover, experiments of promoter mutation and *in vivo *footprinting analysis allowed to show that expression from the *cel6a *promoter is dependent on a CCAAT box bound by the HAP2/3/5 protein complex [78]. The CCAAT motif is found in approximately 30% of the 5'- non-coding regions of eukaryotic genes [79]. In analogy to the mammalian NF-Y complex containing NF-YA, NFYB and NF-YC orthologues of HAP2, HAP3 and HAP5, respectively, they contain a histone fold motif, a structural feature of histones suggesting that NF-Y might be involved in the organisation of the chromatin structure [80]. Thereby the action of acetyltransferases may play a role in the local disruption of nucleosomes since an association of GATA-1 and NF-Y with acetyltransferases p300/CBP has been shown [81, 82]. The corresponding *hap2, hap3 *and *hap5 *genes from *T. reesei* were cloned by Zeilinger *et al.* [83] showing that they encode proteins similar to Hap homologues from other organisms and essential for binding to the CAE (*cbh2*-activating element) in the *T. reesei cel6a *promoter. The HAP2/3/5 complex is considered needed for generating an open chromatin structure required for full transcriptional activation [84]. The hypothesis that the CCAAT sequences in the cellulase promoters could play a conserved role in the generation of an open chromatin structure necessary for full transcriptional activation is supported by the detection of a nucleosome-free region around the XYR1/ACE2/HAP2/3/5- binding area in the *cel6a *promoter, which is flanked by strictly positioned nucleosomes [84]. Induction by sophorose results in a loss of positioning of nucleosomes -1 and -2 downstream of the binding area, thus making the TATA box accessible. A mutation in the CCAAT box shifted this positioning, thus proving the role of the HAP2/3/5 complex in this process [84]. These data provide an experiment based explanation of the advantage for clustering of cellulases in the genome of *T. reesei* and illustrate that chromatin regulation is a suitable target for strain improvement. For instance, it is worth noting that Zou *et al.* [85] have recently demonstrated that replacement of the CREI binding sites within the *cbh1* promoter of *T. reesei* with the binding sites of transcription activator, namely the HAP2/3/5, besides the ACEII, led to improvement of promoter efficiency. The new developped promoter was shown able to induce expression of the green fluorescent protein reporter by 5.5-fold in inducing culture medium and 7.4-fold in repressing culture medium.

ACE1 contains three Cys2His2-type zinc fingers and it was shown to bind *in vitro *to eight sites containing the core sequence 5'-AGGCA scattered along the 1.15-kb *cel7a *promoter [86]. Deletion of *ace1 *resulted in an increase in the expression of all the main cellulase and hemicellulase genes in sophorose- and cellulose-induced cultures, indicating that ACE1 acts as a repressor of cellulase and xylanase expression [87] and of *xyr1 *during growth on D-xylose [72]. A strain bearing a deletion of both the *ace1 *gene and *ace2 *gene expressed cellulases and xylanases similar to the Δ*ace1 *strain, probably due to the remaining activity of XYR1 [87].

All together the above data suggest that the substrate-unspecific activator XYR1 is fine-tuned by more specific transcriptional regulators such as ACE1 and ACE2 (Fig. **1**). This working model concurs with the findings that XYR1 binds to an inverted repeat either as a homo- or a heterodimer, respectively, thereby providing the opportunity for specific regulatory proteins to interact with the accordant promoter and/or XYR1. The role of the HAP2/ 3/5 complex in this regulation may be that of a general transcriptional enhancer raising the accessibility of the other factors to the cellulase promoters.

The putative methyltransferase LaeA is a global regulator that affects the expression of multiple secondary metabolite gene clusters in several fungi, and it can modify heterochromatin structure in *Aspergillus nidulans*. Seiboth *et al.* [88] showed that the expression of genes for lignocellulose degradation are controlled by the orthologous *T. reesei* LAE1: the protein methyltransferase LAE1. In a *lae1* deletion mutant a complete loss of expression of all seven cellulases was observed, auxiliary factors for cellulose degradation, β-glucosidases and xylanases were no longer expressed. Conversely, enhanced expression of *lae1* resulted in significantly increased cellulase gene transcription. Lae1- modulated cellulase gene expression was dependent on the function of the general cellulase regulator XYR1, but also *xyr1* expression was LAE1-dependent. Chromatin immunoprecipitation followed by highthroughput sequencing (‘ChIP-seq’) showed that *lae1* expression was not obviously correlated with H3K4 dior trimethylation (indicative of active transcription) or H3K9 trimethylation (typical for heterochromatin regions) in CAZY (Carbohydrate-Active enZYmes) coding regions, suggesting that LAE1 does not affect CAZyme gene expression by directly modulating H3K4 or H3K9 methylation. These data demonstrate that the putative protein methyltransferase LAE1 is essential for cellulase gene expression in *T. reesei* through mechanisms that remain to be identified.

To learn more about the function of LAE1 in *T. reesei*, Karimi-Aghcheh *et al.* [89] further assessed the effect of deletion and overexpression of *lae1* on genome-wide gene expression. They found that in addition to positively regulating 7 of 17 polyketide or nonribosomal peptide synthases, genes encoding ankyrinproteins, iron uptake, heterokaryon incompatibility proteins, PTH11-receptors, and oxidases/monoxygenases are major gene categories also regulated by LAE1. Chromatin immunoprecipitation sequencing with antibodies against histone modifications known to be associated with transcriptionally active (H3K4me2 and -me3) or silent (H3K9me3) chromatin detected 4089 genes bearing one or more of these methylation marks, of which 75 exhibited a correlation between either H3K4me2 or H3K4 me3 and regulation by LAE1. 

CRE1 is the main transcription factor mediating CCR [90, 91], a mechanism promoting the assimilation of high-energy yielding carbon sources over that of sources yielding less energy, described in more details below. 

### 2.2 Carbon Catabolite Repression of Cellulase and Hemicellulase Genes’ Expression in *T. reesei*

Expression of most of *T. reesei *cellulase and hemicellulases genes does not occur in the presence of glucose in culture medium. Two mechanisms are responsible for this phenomenon: inducer exclusion (that is, inhibition of inducer [= sophorose] uptake) by D-glucose [53] and glucose repression [59, 84, 92]. The latter specifies a transcriptional regulation controlling the preferential use of substrates such as D-glucose or other monosaccharides whose catabolism provides a high yield of ATP namely CCR.

Consequently, one of the earliest attempts for engineering cellulase production was removal of CCR. Classical mutagenesis combined with selection for 2-desoxyglucose resistance (an agent believed primarily to enrich carbon catabolite-resistant mutants) [93] has led to increased cellulase producers such as *T. reesei *RUT C30 [94], RL-P37 [95] and CL847 [96], thus supporting the possible importance of CCR in cellulase formation. 

In *Trichoderma spp.*, the key player in this glucose repression is the Cys2His2 type transcription factor CREI [90, 97]. *cre1* is missing in the cellulase hyperproducer strain RUT C30 [90] and importance of its deletion for the increase of cellulase production has been highlighted recently [98]. The *cre1 *gene is located on scaffold 2: 786955-789433 (ID 120117), and the mutant is characterized by a loss of a 2478-base pair fragment, which starts downstream of the region encoding the CRE1 zinc finger and reaches into the 3'-non-coding region [99]. Le Crom *et al.* [100] discovered that in Rut-C30, in addition to the 29 genes deleted during the generation of NG14, the truncation of *cre1* gene and the frameshift in glucosidase II, nearly 45% of the genes mutated encode transcription factors, components of nuclear import, mRNA metabolism, protein secretion, and vacuolar sorting.

The knowledge of mutations in the hyperproducer *T. reesei* strains was widened by Vitikainen *et al.* [101], reporting an aCGH (Array-Comparative Genomic Hybridization) analysis of the high-producing strains QM9123, QM9414, NG14 and Rut-C30. These authors showed that the 85 kb deletion is not responsible for the high ability of cellulase producing in Rut-C30.

*In vivo *functionality of the CRE1 binding sites has been shown for the *cbh1 *and *xyn1 *promoters of *T. reesei *where mutations in the binding sequences led to constitutive expression of these genes in the presence of D-glucose [92, 102]. Functional CREI binding sites have been shown to consist of two closely spaced 5'-SYGGRG motifs, and it has been suggested that direct CREI repression would occur only through such double binding sites. Phosphorylation of a serine in a conserved short stretch within an acidic region of *T. reesei *CREI has been demonstrated to regulate its DNA binding [103]. Phosphorylation of this serine may involve a casein kinase 2. Casein kinases of this class are known from various other organisms to play a role in the regulation of a large number of transcription factors [104]. However, the SNF1 kinase, which plays a central role in the regulation of CCR in yeasts [105], appears not to be involved in the phosphorylation of CRE1 in *T. reesei *[106].

Another gene whose product is involved in CCR in *T. reesei *is represented by* cre2 *whose disruption led to deregulation of genes normally subjected to CCR [107]. Interestingly, the E3 ubiquitin ligase LIM1 also responds to cellulase inducing conditions and binds to the *cbh2*-promoter [108]. 

The way in which the presence of glucose triggers CCR is still only poorly understood in filamentous fungi. In *S. cerevisiae*, the D-glucose and D-fructose phosphorylating enzymes are also involved in D-glucose and carbon catabolite sensing, due to the presence of three hexose-6-phosphorylating enzymes including two hexokinases and one glucokinase. Each of them enables *S. cerevisiae *to grow on D-glucose, but the hexokinase Hxk2p is responsible for the main enzymatic activity and glucose repression mediated by the carbon catabolite repressor Mig1p (whose DNA-binding domain is highly similar to that of CRE1) [109-111]. The mechanism by which Hxk2p contributes to glucose repression has not yet been fully elucidated, but its catalytic activity seems to be dispensable and thus signal transmission may rather depend on substrate binding-induced conformational changes in the Hxk2p protein or a direct regulatory role of the Hxk2p in the nucleus (discussed, for example, in Linhoff *et al.* [80]). 

Portnoy *et al.* [112] investigated how *xyr1, ace1* and *ace2* are regulated in cellulase induction conditions and how this regulation relates to carbon catabolite repression in the low cellulase producer strain* T. reesei *strain QM 9414, the high-producer strain RUT C30 [94, 113] and the hyperproducer strain *T. reesei *CL847 [96]. They demonstrated that in QM 9414 all three genes are induced by lactose and *xyr1 *is also induced by D-galactose. Moreover, *ace1 *is carbon catabolite repressed, whereas full induction of *xyr1 *and *ace2 *requires CRE1. These regulatory patterns showed significant differences in RUT C30 and CL847 strains. Rate of cellulase production by strain CL847 on lactose was around 15-fold higher than that for strain QM 9414, consistently with the 15-fold-increase of the *cbh1* transcript level. These data indicate that gene expression is a major limiting step for cellulase biosynthesis. Consistent with its role as the major transcriptional regulator of cellulase gene expression, a strongly increased basal expression of *xyr1* was observed in strain CL847, which was further induced by lactose. This increase indicates an improved function of the transcriptional machinery required for *xyr1* expression in strain CL847. The basal expression of *ace2* was not significantly altered in strain CL847, and the inducible level was the same as that in strain QM 9414. This indicates that the lack of CRE1 function, which seems to be required for *ace2* gene expression, as indicated by the lower expression levels in the Δcre1 mutant, has been overcome during the breeding of CL847. While these data suggest that *ace2* expression is not limiting for cellulase induction on lactose, they nevertheless show that wild-type expression levels appear to be necessary for the formation of high levels of cellulase. Expression of *ace1*—even though it is a repressor of cellulase formation—was also increased in the mutant strain CL847. However, *ace1* is subject to CRE1-dependent CCR. The comparison reveals that the basal expression level of *ace1* in CL847 is lower than that in the Δcre1 strain and decreases during the glucose feed. The approximate doubling of this level during the lactose feed is conserved, however. It has therefore been concluded that carbon catabolite derepression of *ace1* has partially reverted in CL847, leading to a lower concentration of this repressor under cellulase-producing conditions. The present findings of reduced *xyr1* but increased *cbh1* transcription in the Δcre1 strain would be consistent with the operation of post-translational modification of XYR1. Nevertheless, these data show clearly that the expression of *xyr1*, *ace1*, and *ace2* has been significantly altered in the hyperproducer CL847, suggesting that their wild-type expression was insufficient for hyperproduction. Identification of the proteins and genes responsible for the mechanisms observed may result in a major breakthrough in the understanding of cellulase formation and may offer a straightforward means for its improvement. These observations suggest that a strongly elevated basal transcription level of *xyr1 *and reduced upregulation of *ace1 *by lactose may have been important for generating the hyperproducer strain and that thus, these genes are major control elements of cellulose production.

## 3 REGULATION OF PRODUCTION OF CELLULASES AND HEMICELLULASES IN *NEUROSPOSRA CRASSA*

*Neurospora crassa*, a non pathogenic filamentous fungus of the class ascomycetes, is a well-known model organism that has been used for 90 years to study genetics, biochemistry, and fungal biology [114]. It was genetically characterized [115] and it has been shown to be able to degrade cellulose since 30 years ago [116]. *N. crassa* is able to synthesize and secrete high levels of all three enzyme types involved in cellulose degradation [117-121], as well as endoxylanase and β-xylosidase activities [122, 123]. There are 23 predicted cellulase genes and 19 predicted hemicellulase genes in the genome of *N. crassa *(*http://www.broadinstitute.org/ annotation/genome/neurospora/MultiHome.html*). In addition, *N. crassa* is a well-known ethanol producing microorganism that has been used for fermentation of agricultural residues [124]. 

The cellulase complex in *N. crassa* is composed by four endoglucanases, three exoglucanases and one β-glucosidase [125]. A summary of inducibility of cellulases, hemicellulases and related enzymes in *N. crassa *is reported in the Table **1**. In 1964, Eberhart *et al.* [126] showed the presence of two β-glucosidases, including an aryl-β-glucosidase and a cellobiase, acting complementarily in *N. crassa*. Based on their production in response to specific inducers or various conditions of growth, these enzymes represent two fundamentally different classes of disaccharidases. Results of Eberhart *et al.* [127] on the induction of β-glucosidases (EC 3.2.1.21) in *N. crassa*, showed that the aryl-β-glucosidase can be induced either by disaccharides, that are usually used as substrates by this class of enzyme, or by monosaccharides that are not their usual substrates [120, 128-132]. Induction in the presence of monosaccharides and spontaneous production associated with conidiation is reversal of catabolite repression [133-135] because enzymes are not produced when a significant level of glucose is present in the induction medium. Both cellobiase and aryl-β-glucosidase seem to be exceptions to the general situation that disaccharide substrates are not the best inducers of specific disaccharidases in *Neurospora* [120, 130, 132], being production of both enzymes induced by cellobiose. Aryl-β-glucosidase production is semiconstitutive at late stages of culture growth prior to conidiation. At early stages, aryl-β-glucosidase is induced by cellobiose, laminaribiose, and gentiobiose, and in small part induced by galactose, amino sugars, and aryl-β-glucosides. Among the monosaccharides, xylose and galactose induce aryl-β-glucosidase. Cellobiase is induced by cellobiose, but other inducers have little effect on this enzyme such as galactose and maltose. Cellobiase activity is very low in all stages of the vegetative life cycle in the absence of β-glucoside inducer. Experimental results showed that a mixture of xylose and cellobiose induced increase of cellobiase, while added xylose did not change significantly the induction of aryl-β-glucosidase. Cellobiose is clearly the best inducer, with an optimum effect from 0.05 to 1 mM. The induction of β-glucosidases was inhibited by glucose, 2-deoxy-D-glucose, and sodium acetate. Sodium phosphate concentrations between 0.01 and 0.1 M stimulated induction of both enzymes, while concentrations above 0.1 M were inhibitory. The optimal condition for induction of both β-glucosidases was pH 6.0. Cellobiase induction was relatively more inhibited than aryl-β-glucosidase in the range of pH 6.0 to 8.0. The time required for the induction of these enzymes by cellobiose is 6 hours. As described below, fatty acids and surfactants have positive effects on the cellulases production [121], however, experimental results showed that oleic acid had no effect on production of β-glucosidase, while Tween 80 decreased its production [121]. This is probably due to some difference in the cellulase and β-glucosidase released. In fact in most organisms studied, β-glucosidase is an intracellular enzyme, released only by autolysis [136]. A recent study [137] demonstrated that a *N. crassa* mutant carrying deletions of two genes encoding extracellular β-glucosidase enzymes and one intracellular β-glucosidase lacks β-glucosidase activity, but its cellulase gene expression is efficiently induced in the presence of cellobiose, cellotriose, or cellotetraose as a sole carbon source while sophorose does not act as an inducer. Furthermore, the inclusion of a deletion of the catabolite repressor gene, *cre-1*, in the triple β-glucosidase mutant resulted in a strain that produces higher concentrations of secreted active cellulases on cellobiose. So cellobiose is an inducer of β-glucosidases but carbon catabolite repression (CCR, see the following paragraph) masks this inducing activity.

Eberhart *et al.* [116] studied the extracellular endocellulase (EC 3.2.1.4) production in mycelia and ungerminated conidia of *N. crassa*. They demonstrated a simple induction system of cellobiose and potassium phosphate buffer (pH 6.0) of extracellular cellulase to provide energy and substrates for protein synthesis. Yazdi *et al.* [125, 121, 138] have shown for the first time that *N. crassa* is capable of synthesizing and secreting high levels of the cellulase complex enzymes growing on microcrystalline cellulose, and other carbon sources, as an inducer for the enzymes. They also studied the role of surfactants and fatty acids on the production of the cellulases, since in other species it has been reported that surfactants and fatty acids stimulate production of the cellulase complex [139-141]. Yazdi *et al.* [121] demonstrated that the presence of C18 fatty acids and surfactants, such as Tween 80, increases production of both endoglucanase and exoglucanase in the medium. It is probably due to an increase in the permeability of the cell membrane, thus permitting more of the enzymes to be secreted, as postulated for other species by Reese & Maguire [139] and Demain & Birnbaum [142].

Analysis of the effects of different carbon sources, such as glucose, xylan and cellulose, on the production of extracellular cellulases and xylanases by *N. crassa*, reported by Mishra *et al.* [122], showed that the extracellular activities were very poor when the fermentation was carried out with glucose, while the maximum xylanase production was observed when *N. crassa* was grown on commercial xylan. However, significant amounts of xylanase were also produced when cellulose powder was used as a carbon source.

In 2012, Sun *et al.* [143] showed that *N. crassa* responds to the presence of cellulose (Avicel) by inducing both cellulase and hemicellulase gene expression, while the exposure to xylan only induces hemicellulase gene expression. In addition, exposure to Avicel induces some hemicellulase genes to a much higher expression level than exposure to xylan. These data suggest crosstalk between inducer molecules and regulatory pathways that are involved in deconstruction of plant cell walls in filamentous fungi. In this work, 353 genes have been identified that were significantly induced by xylan; in particular three genes, *gh51-1 *(arabinofuranosidase), *gh10-2 *(endoxylanase) and *gh43-5 *(β-xylosidase) showed increased expression levels of over 200 fold. Among 353 identified genes only 30 genes were induced by exposure to xylose, although none of the xylanolytic-related genes was essential for growth of *N. crassa* on xylan, in fact mutations in a number of them affected xylanase activity. These observations indicate some redundancy among enzymes associated with hemicellulose degradation, similar to those identified with cellulose degradation [48].

### 3.1 Transcriptional Factors Involved in Regulation of Cellulase and Hemicellulase Genes Expression in *N. crassa*

The knowledge of *N. crassa* genome sequence [144] has allowed the identification of the proteins involved in regulation of cellulase ad hemicellulase genes expression (Table **2**, Fig. **1**). 

Two zinc binuclear cluster transcription factors (CLR-1 and CLR-2) are important regulators of genes encoding both cellulases and hemicellulases in the presence of cellulose as carbon source, but they are not required for growth or hemicellulase activity production in the presence of xylan as reported by Coradetti *et al.* [145]. In particular, Coradetti *et al.* [145] demonstrated that CLR-1 is a crucial element in cellobiose sensing mechanism of *N. crassa* during its growth on avicel. CLR-1 promotes expression of several genes necessary for cellobiose utilization, as well as that of *clr-2*. CLR-2, maybe in a complex with CLR-1 directly induces cellulase and hemicellulase gene expression, when *N. crassa* is grown on avicel. Phylogenetic analyses of CLR-1 and CLR-2 protein sequences performed by the same group, showed that these factors are conserved in the genomes of most filamentous ascomycete fungi degrading cellulose suggesting that homologs of CLR-1 and CLR-2 play an important role in plant cell-wall degradation.

In nature, the enzymatic breakdown of plant cell wall polymers can occur in different surrounding pHs and there is a regulatory mechanism controlling pH-dependent transcriptional regulation. *pacc* gene in *N. crassa* (ORF NCU00090) is the *pacc*/RIM101 orthologue, extensively studied in *A. nidulans* and *S. cerevisiae* [146]. The transcription factor PacC responds to changes in extracellular pH by activating specific alkaline genes and repressing specific acid genes [147, 148].

A xylan degradation regulator-1 (*xlr-1* NCU06971) is essential for hemicellulose degradation in *N. crassa*. *xlr-1* encodes a member of a TF family containing conserved fungal Zn(2)-Cys(6) binuclear cluster domain with significant amino acid homology to *xyr1* in *Trichoderma *species [148], sharing 57.6% identity with homolog in *T. reesei*. Recently, Sun *et al.* [143] demonstrated that a deletion of *xlr-1*gene abolishes growth of *N. crassa* in both xylan and xylose containing media, but it slightly affects the growth on Avicel and the production of cellulase activity in the presence of this substrate. To determine regulatory mechanisms for hemicellulose degradation, the authors explored the transcriptional regulation of XLR-1 under xylose, xylanolytic and cellulolytic conditions. Their results showed that XLR-1 regulates only some predicted hemicellulase genes in *N. crassa* and was required for a full induction of several cellulase genes. Moreover, among the genes induced by xylan there are 19 permease/transporter genes and their full induction requires a functional *xlr-1*. Of these 19 transporters, five have been functionally tested for transport of D-glucose and D-xylose. Another transcription factor identified in *N. crassa* was NIT2 protein (AreA in *A. nidulans*), a member of the GATA factors family, characterized in *N. crassa* as a positive regulator of genes encoding enzymes for nitrogen source catabolism under nitrogen limiting conditions [149, 150]. Goncalves *et al.* [148] suggested that *nit-2* also acts as a repressor of carbon metabolism. They analyzed *cis *elements present in the gene encoding glycogen synthase (*gsn*) promoter and showed that *nit-2* is able to bind these *cis *elements. Moreover, the knocked-out *nit-2* strain showed loss of glycogen accumulation despite having low *gsn *gene expression as compared to the wild-type strain, suggesting they may have a role in glycogen metabolism regulation. A link between carbon and nitrogen regulation was already reported by Lockington *et al. *[151] in *A. nidulans*. Although the result was preliminary, the authors suggested the existence of a link in the regulation of the carbon and nitrogen utilization pathways in filamentous fungi.

### 3.2 Carbon Catabolite Repression of Cellulase and Hemicellulase Genes Expression in* N. crassa*

Sun *et al.* [152] investigated CCR of cellulase expression in *N. crassa *and they showed that, under cellulolytic conditions, CRE-1 regulates genes involved in plant cell wall utilization by directly binding to adjacent motifs in promoter regions and also may compete for binding with positive regulatory factors. They demonstrated that deletion of *cre-1* caused constant expression of cellulase genes, resulting in higher cellulolytic enzyme activity. Moreover, *cre-1* caused the repression of cellulolytic genes during growth on Avicel. Some genes known to be directly regulated by CRE-1 homologs in other systems (such as *cbh-*1of *T.reesei* and XlnA of *A. nidulans*) and also a large number of other target genes of predicted or unknown function in *N. crassa* were identified. These genes may be regulated directly or indirectly by CRE-1. For example, CRE-1 binds to the promoter region of *cbh-1* in *N. crassa* and may compete for binding with pathway specific cellulolytic regulator required for induction; the identity of cellulolytic regulators in *N. crassa* is currently unknown. Among CRE-1 targets identified in *N. crassa* there are a hypothetical protein of unknown function (NCU 03181), an additional xylanase (NCU07225) and *gh6-3* (NCU07190). It is worth of note that a MFS monosaccharide transporter (NCU04963) was identified as a direct target of CRE-1 in *Neurospora*. These results suggest that CRE-1 may directly regulate genes involved in sugar transport, in addition to regulating genes encoding regulatory/enzymes associated with utilization of alternative carbon sources. In summary, CRE-1 functions as a global transcription factor in *N. crassa* and affects both gene repression and activation, both directly and indirectly. 

The same authors recently [143] studied the *cre-1* regulation in hemicellulase expression. As described above, in *N. crassa*, transcription of most hemicellulase genes is via induction by xylanolytic molecules and is regulated via *xlr-1* and/or other transcription factors. However, the hemicellulolytic system is also responsive to CCR. CRE-1 mediated CCR regulates the expression level of some, but not all, hemicellulase genes in *N. crassa* under Avicel conditions. *xlr-1* is regulated by a combination of induction and derepression and it is also subjected to non-CRE-1 mediated CCR. These observations imply that other mechanisms regulate CCR in filamentous fungi in addition to CRE-1, similar to what has been described for *S. cerevisiae* [153].

## 4 REGULATION OF PRODUCTION OF CELLULASES AND HEMICELLULASES IN *ASPERGILLUS* SPP

The genomes of four *Aspergillus spp.*, *A. nidulans*, *A. oryzae*, *A. niger* and *A. fumigatus*, have been recently sequenced (*http://www.aspergillusgenome.org/*), and shown to contain around 200 genes -out of 14,600- involved in polysaccharides’ degradation [154]. *Aspergillus spp*. have been so far described as high cellulases’ producers and many genes coding for cellulase, endoxylanases, β-xylosidases and pectinases have been cloned and characterized from *Aspergillus* spp. strains [24]. A summary of inducibility of cellulases, hemicellulases and related enzymes in *Aspergillus spp.* is reported in the Table **1**.

The influence of carbon and nitrogen sources on the production of cellulases has been so far investigated showing that the enzyme production is strongly variable according to the carbon source.

For instance, Hanif *et al.* [155] showed that even low concentrations of glucose negatively affect β-cellobiohyd rolase (CBH) production in *Aspergillus niger*, whilst cellulose and wheat bran stimulate β-cellobiohydrolase and filter paperase (FPase) activities, respectively. It was shown that addition of glucose inhibited cellulase production, even in cultures of *A. niger* gowing on wheat bran, shown to be a good inducer. 

In several manuscripts, lactose has been defined as the best inducer for cellulase production in *Aspergillus* spp. Mrudula and Murugammal [156] confirmed that lactose is the best inducer of cellulase activity production by *Aspergillus* *niger*. In fact, lactose was shown the best carbon source to obtain high level of both CMCase and FPase activities, in both liquid and solid state fermentation.

As shown by Ali and Sayed [157], xylose is the best carbon source for induction of both endo- and exo-cellulase activity production in *A. terreus*, whilst the production of β-glucosidase is positively affected by both glucose and xylose. Lignocellulosic substrates, like agroindustrial wastes, have been so far described as good substrate for cellulase and xylanase production by filamentous fungi, as *Aspergillus* spp. Among the several examples, Ghori *et al.* [158] recently demonstated the properties of corn-stover as an inducer of cellulase activity production by *A*. *niger*. Moreover, they demonstated that addition of cane molasses and yeast sludge to the fermentation medium leads to an increase of cellulase production. However, the induction mechanism involved in solid state fermentation have been shown to be more complex [159].

The effect of several carbon sources on glycosyl hydrolases gene expression has been studied by Nazir *et al.* [160] who reported differential expression of endoglucanase and beta-glucosidase isoforms of *A. terreus*, in both solid and liquid cultures. Maximal expression of four endoglucanase isoforms was observed in presence of rice straw and corn cobs, in solid state and liquid fermentation, respectively. Addition of fructose and cellobiose to corn cobs containing medium caused the up-regulation of endoglucanase activity, whereas addition of mannitol, ethanol and glycerol selectively repressed the expression of at least three endoglucanase isoforms. As far as the beta-glucosidase profiling is concerned, addition of glucose, fructose, sucrose, cellobiose, mannitol and glycerol resulted in down-regulation of most of the isoforms.

Many manuscripts have been reported concerning induction of xylanase production in *Aspergillus* spp.. It is well known that xylose, xylan and crude xylan-containing substrates mainly induce xylanolytic enzymes production in *Aspergilli *spp*.*. There are rare cases where other monomeric or polymeric substrates, such as glucose and cellulose, induce xylanolytic expression. For instance, Hrmova *et al.* [161] observed the induction of xylanolytic enzymes by cellulose, cellobiose and even by a heterodysaccharide consisting of glucose and xylose in *A. terreus*. The regulation of xyalonlytic enzymes is not identical in all *Aspergillus* spp.. As a matter of fact, Kimura *et al.* [162] cloned a xylose-inducible, glucose repressed endoxylanase gene from *A. oryzae* in *A. nidulans*, where its expression was instead increased by adding glucose. 

Pinaga *et al.* [163] studied the effect of several compounds on xylanase production by *A. nidulans*. Xylooligosaccharides such as xylobiose, xylotriose and xylotetraose induced xylanase activity production, their efficiency being directly related to their chain length. However, xylans such as wheat arabinoxylan, oat spelt xylan, birchwood xylan and 4-O-methyl-D-glucorono-D-xylan were found to be the most powerful inducers. Xylose, on the contrary, was not shown to be a good inducer.

Xylanases production by *A. phoenicis *was shown positively affected by xylan, xylose and *b*-methylxyloside, similarly to the cases of other fungi belonging to the *Aspergillus* genus such as *Aspergillus sydowii *and *A. tubingensis*, as studied and discussed by Rizzatti *et al.* [164]. This study also demonstrated that the levels of production of xylanase by *A. phoenics*, decreased when glucose was added to the inducers xylan or xylose, similarly to *A. sydowii* whose xylanase production is inhibited by glucose [165].

Methyl β-d-xyloside was shown a more effective inducer than xylan, for both extracellular xylanase and intracellular β-xylosidase by Simao *et al.* [166]. The same group also demonstated that both glucose and cycloheximide inhibit the positive effect of methyl β-d-xyloside on xylanase production. However, not much is known about the uptake system for the inducers xylose and xylobiose in *Aspergillus spp,.* but more is known about the formation of the inducing compounds. For instance, the *A.niger *β-xylosidase, encoded by *xlnD*, has been shown to have an important role in xylanolytic inducer formation, being active towards xylan and xylooligosaccharides for the formation of D-xylose [167]. 

Galacturonic acid is the main inducer of several pectinolytic enzymes encoding genes such as *pelA, plyA, pgaX*, *rglA *and* pmeA* [168]. As reported by de Vries *et al.* [169], galacturonic acid positively affects even the expression of several genes encoding enzymes which act on the pectin side chains such as arabinofuranosidases (*abfA *and *abfB*), endoarabinase (*abnA*), endogalactanase (*galA*) and galactosidase (*lacA*). 

As far as the induction of extracellular arabinases is concernd, pentose sugars and polyols generated by the metabolic pathway of L-arabinose and D-xylose catabolism were shown to be invooved in *Aspergillus niger *arabinases production*. *Particularly, induction occurred with L-arabinose and L-arabitol but not with D-xylose or xylitol, L-arabitol being the best inducer for a-L-arabinofuranosidase and endo-arabinase activities [170].

### 4.1 Transcriptional Factors Involved in Regulation of Cellulase and Hemicellulase Genes Expression in *Aspergillus* spp

Transcription factors involved in the regulation of *Aspergillus* spp. (hemi)cellulolytic enzymes encoding genes, mostly belong to Zn(II)2Cys6 binuclear cluster DNA-binding motif family (Table **2**, Fig. **1**).

XlnR is the main transcriptional activator which has been largely studied for its involvement in the regulation of cellulases, hemicellulases and accessory enzyme genes for xylan degradation in *Aspergillus* spp.. It is an orthologue of the *xyR1 *gene of *T. reesei *[18]. 

van Peiji *et al.* [171] finely described the role of XlnR transcriptional activator. It has been demonstrated that XlnR regulates the transcription of the *xlnB*, *xlnC* and *xlnD *genes encoding endoxylanases B, endoxylanase C and β-xylosi dase, respectively. It is also involved in the activation of cellulase genes transcription, such as those coding for the two endoglucanases *eglA *and *eglB*. In addition, XlnR has been shown to positively affect the transcription of several accessory enzymes gene involved in hemicelluloses degradation, including glucuronidase A, acetylxylan esterase A, arabinoxylan arabinofuranohydrolase A and feruloyl esterase A. 

Several northern blot analyses have been performed on *A. niger* strains, in order to demonstrate the important role of XlnR on the activation of different glycosyl hydrolases genes transcription. These analyses allowed the comparison of level of expression of genes of interest in an *A. niger *wild-type strain, a *xlnR *loss-of-function mutated strain and a multiple-copy strain [171, 172]. 

More recently, similar studies have been performed by Tani *et al.* [173] who demonstrated that cellulose affects positively both cellulase and hemicellulase activities production in *A. aculeatus*, through two different pathways, namely XlnR-dependent and XlnR-independent pathways. Real-time PCR (Polymerase Chain Reaction) experiments have been performed to identify the genes controlled by the XlnR-independent pathway. Particularly, both cellobiose and cellulose were shown to induce the expression of the gene regulated by XlnR-independent signaling pathway, the latter stimulating expression of FIII-avicelase (*cbhI*), FII-carboxymethyl cellulase (*cmc2*), and FIa-xylanase (*xynIa*).

Recently, further analyses on (hemi)cellulase genes regulation in *A. aculeatus* have been performed by Kunitake *et al.* [174]. ClbR, a new activator with a Zn(II)2Cys6 binuclear cluster DNA-binding motif specific for fungi, has been identified. It has been shown to control the cellobiose and cellulose responsive induction of cellulase and xylanase genes which are regulated by both XlnR-dependent and XlnR-independent signaling pathways. For instance, disruption of *clbR* gene caused the decrease of the cellobiose- and cellulose-responsive induction of the *cbhI*, *cmc2*, and *xynIa* genes and the cellulose-responsive induction of the *cmc1* and *xynIb* genes Kunitake *et al.* [174].

Differently from *T. reesei*, fine-tuning transcription factors like Ace1 and Ace2 cannot be found in *Aspergillus* spp. The putative ACEI proteins of *A. nidulans*, *stzA* (AF202995), is deposited into the database as a gene encoding a protein that alleviates sensitivity to salt and DNA damaging agents. Interestingly, *stzA* has been identified as an orthologue of the *T. Reesei* ACE1 gene [175]. The authors provided evidence of competition, or interaction, between the ACE1/StzA and AreA binding sites in promoters of *stzA *and its orthologs, and in genes involved in the metabolism of amino acids. The *A. nidulans *and *A. fumigatus cpcA *(cross pathway control regulator of amino acid biosynthesis) promoters have seven potential ACE1/StzA binding sites, six of which are highly conserved in position. The presence of potential CPC1 binding sites (5'-TGAC/GTCA) in the *stzA *and *ace1 *promoters suggests an intriguing link between intracellular amino acid availability and cellulase gene expression. In accordance with these findings, a recent study by Gremel *et al.* [108] indeed revealed that cellulase gene expression can be enhanced by the addition of methionine.

PacC is the major factor involved in pH-dependent expression in *Aspergillus spp.. *pH regulation of genes encoding cell wall-degrading enzymes has not been studied in detail in *Aspergillus*. However, indications for pH-dependent expression of xylanolytic and pectinolytic genes have been obtained [176]. Kojma *et al.* [176] demonstrated that *A. kawachii *produces different polygalacturonases using culture media with different pHs whilst *A. nidulans* PacC mutant strain does not produce arabinofuranosidase activity [177] and two endoxylanase, *xlnA *and *xlnB* [178]. Even cellulase production in* A. fumigatus *is affected by pH [179]. Indeed, two and one PacC *consensus* sites have been revealed in the promoter regions of *xlnA, xlnB *[180] and* xlnD *[181], respectively. The role of the transcriptional activator AreA on total cellulase production has been studied by Lockington *et al.* [182]. *areA* gene product is known to control the expression of genes encoding the enzymes involved in nitrogen metabolism in ammonium derepressing conditions [183]. The homologous of AreA was identified in *N. crassa* as NIT2 protein, [149, 150].

It has been shown that the amount of total secreted cellulase activity increased in a strain containing the constitutively activating *areA* allele, xprD1, and decreased in a strain containing the loss of function allele, *areA217*. To deepen AreA role in cellulase genes regulation, two genes encoding exocellulases, and one gene encoding an endocellulase were cloned. The putative regulatory regions of all the genes contain potential binding sites for the global carbon and nitrogen regulatory proteins, CreA and AreA, potential *consensus* binding sites for XlnR, whilst the AceII DNA binding *consensus* sequence involved in induction in *T. reesei*, misses in all the genes [182]. Real-time PCR techniques were used to assess the relative expression levels of genes encoding hydrolase activities and of the genes encoding regulatory elements such as AreA, PacC and CreA in an effort to identify possible transcriptional regulation mechanisms in *A. oryzae* solid state fermentation [159]. This study showed the complexity of the regulation of genes coding for hydrolytic enzymes under solid state fermentation, as other factors such as post-transcriptional regulation appeared to be involved.

### 4.2 Carbon Catabolite Repression of Cellulase and Hemicellulase Genes Expression in *Aspergillus* spp

It has been reported that *creA*, *cre B* and *creC* genes products are involved in the regulatory mechanism of carbon catabolite repression in *Aspergillus spp.* [183-187]. CreA-mediated repression in *Aspergillus *has been demonstrated for genes encoding cellulase, arabinases, several endoxylanases and other xylanolytic activities such as xylosidase, feruloyl esterase and some pectinases [22]. The binding *consensus* motif for *A. nidulans *CreA was determined to be 5'-SYGGRG [188]. Besides glucose, other monomeric carbon sources result in CreA-mediated repression of gene expression, such as xylose. For instance, high concentrations of xylose have been shown to activate the CreA-mediated repression, by down-regulating the expression levels of several xylanolytic and cellulolytic genes in *A. niger* [169] and *A. terreus* [157].

Interestingly, Flipphi *et al.* [189] showed that in *A. nidulans*, mutations in both the single glucokinase and the single hexokinase genes belonging to the fungus, lead to a CreA-mediated carbon catabolite derepression, similarly to *T. reesei *which also features only one glucokinase and one hexokinase.

CreB encodes a deubiquitinating enzyme and it is a functional member of a novel subfamily of the *ubp *family defined by the human homolog UBH1 [186]. It forms a complex with a WD40-repeat protein encoded by *creC *[182], which is required to prevent the proteolysis of CreB in the absence of CCR [187]. Interestingly, the E3 ubiquitin ligase LIM1 also responds to cellulase inducing conditions and binds to the *cbh2*-promoter [108].

In addition, CreD has been reported to be involved in CCR of *Aspergillus* sp.. Mutations in *creD *suppress the phenotypic effects of mutations in *creC *and *creB *[190]. CreD contains arrestin domains and PY motifs and is highly similar to *S. cerevisiae *Rod1p and Rog3p, which interact with the ubiquitin ligase Rsp5p [191].

## 5 CONCLUSIONS

The regulation of (hemi)cellulolytic genes appears to be basically the same among filamentous fungi such as *T. reesei,* *N. crassa,* *Asperigillus* spp., although their regulatory mechanisms are quite complex and present some differences. In particular, cross talks between expression of cellulolytic and hemicellulolytic genes make the regulatory mechanisms more complicated. Xyr homologs (Xyr1 of *T.reesei*, XlR of *N. crassa,* and XlnR of *Aspergillus* spp) mediate expression of both xylanolytic and cellulolytic genes in response to xylan. Moreover, Xyr homologs mediate cellulose-inductive expression of the xylanolytic genes as well as the cellulolytic genes. In addition to Xyr homologues, AceI and AceII in *T. reesei,* and ClbR in *A. aculeatus*, are suggested to be involved in the regulation of the expression of these genes. This further makes the regulatory mechanisms of (hemi) cellulolytic genes complicated. Unfortunately, the roles of AceI and AceII in the regulation of cellulolytic genes remain ambiguous and need to be investigated further. Carbon catabolite repression of cellulase expression appears to be essentially the same among fungi. As described above, major inductive signals for fungi to degrade plant cell wall are derived from cellulose and xylan. Xyr homologs receive inducing signals from these two different polysaccharides and activate transcription of many (hemi)cellulolytic genes. Xyr homologues could recognize two distinct inductive signals, in response to cellulose and xylan, prior to induction of the target genes due to their differential conformational changes.

As a main difference among the cellulase and hemicellulase regulatory systems from different fungi, these systems appear to be more specialized in *T. reesei* than in the other fungi, considering that *T. reesei* fine-tuning transcription factors like Ace1 and Ace2 cannot be found in *Aspergillus* and *Neurospora* spp.

However, more research is needed to completely disclose the molecular mechanisms of regulation of cellulase and hemicellulase gene expression in *T. reesei*, *Aspergillus* and *Neurospora* spp.. Their elucidation could provide a basis for the rational application of transcriptional regulators for biotechnological processes in filamentous fungi, leading to efficient bioethanol production from lignocellulosic biomass.

## Figures and Tables

**Table 1.** Inducibility of Cellulases, Hemicellulases and Related Enzymes in *T. reesei, N. crassa* and *Aspergillus spp. X: repressor;* +*: inductor; -: no action*

Substrate	Glycerol	Glucose	Sorbitol	Cellulose	Cellobiose	Xylose	Sophorose	Lactose	Xylobiose	Galactose	Laminaribiose	Gentiobiose	Aryl- β-glucosides	Maltose	Xylan	GalacTuronic acid	Fructose	Mannitol	Arabinose	Arabinitolo	Mannose	Sorbose	Twen 80	C18 Fatty acids	References
Enzyme																									
*T. reesei*																									
bgl1					+	+	+		+																[24]
bgl2						+	+		+																[48]
bxl1						+	+		+																[48]
cbh1	-		-	+	+	+	+	+							+										[51, 59]
cbh2	-		-	+	+	+	+		+																[59]
cel5b	+	+					+	+																	[54]
egl1	-		-	+			+																		[51, 59]
egl2	-		-	+			+																		[59]
egl3							+															+			[192]
egl5	-		-	+			+																		[59]
xynI		x			+	+	+		+						+							+			[192]
xynII		x			+	+	+		+						+							+			[192]
xyn3						-	+															+			[192, 193]
a-AF																						+			[192]
abf1		x																	+	+					[71]
abf2		x																	+	+					[71]
abf3		x																	+	+					[71]
agl1									+	+															[24]
agl2									+	+															[24]
glr1									+																[24]
axe1										+															[24]
***N. crassa***																									
Endocellulase					+																		+	+	[121]
β-glucosidases																							x	-	[121]
aryl-β-glucosidase		x			+	+				+	+	+	+												[127]
Cellobiase		x			+	+				+				+											[127]
Xylanase		-		+											+										[122]
xyr-1						+																			[143]
xdh-1						+																			[143]
xyk-1						+																			[143]
Cellulase				+											-								+	+	[121]
***N. crassa***																									
gh 43-2				+											+										[143]
gh 51-1															+										[143]
gh 10-1															+										[143]
gh 43-5				+											+										[143]
arabinase															+										[143]
gh 11-2				+											+										[143]
gh 10-2				+											+										[143]
gh 53-1				+											+										[143]
gh 1-1						+																			[143]
β-galactosidases						+		+		+															[199]
gh5-1				+																					[143]
gh 61-7				+																					[143]
gh 61-4				+																					[143]
gh 61-1				+																					[143]
gh 61-3				+																					[143]
gh 61-6				+																					[143]
gh 7-1				+																					[143]
gh 6-3				+																					[143]
gh 61-13				+																					[143]
gh 61-5				+																					[143]
gh 11-1				+																					[143]
gh 74-1				+																					[143]
gh 10-1				+																					[143]
***Aspergillus spp.***																									
Endoglucanase	x			+													+	+							[160]
β-glucosidase	x			+													+	+							[160]
Xylanolytic enzymes		+		+		+									+										[20, 161]
eglA/B					+	+																			[161, 200]
pelA/B/C/D/E/F																+									[168, 200]
plyA																+									[168, 200]
pgaA/B/C/D/E																+									[168, 200]
***Aspergillus spp.***																									
pgaX/I/II																+									[168, 200]
pmeA																+									[168, 200]
rglA																+									[168, 200]
afbA																+			+	+					[24,169,170]
afbB																+			+	+					[24,169,170]
abnA																+									[168,169,200]
lacA						+				+						+			+		+				[168,169,200]
aguA						+										+									[169, 200]
axeA						+																			[169, 200]
faeA						+																			[169, 200]
axhA																			+	+					[169, 200]
rhgA/B																+									[169, 200]
axhA						+										+		+							[24]

**Table 2.** Positive and Negative Regulators of Expression of Genes Coding for (hemi)cellulolytic Enzymes and Their Binding *consensus* Sequences in the Target Promoters

*Trichoderma reesei*			
Positive Regulators			
Name	Structure	Consensus Region	References
XYR1	Zinc binuclear cluster protein	5’-GGCTAA	[194] [25]
ACE2	Zinc binuclear cluster proteins	5ʹGGCTAATAA	
HAP2	Multimeric protein complex	5’- CCAAT	
HAP3			
HAP5			
**Negative regulators**			
**Name**	**Structure**	**Consensus region**	**References**
ACE1	Three Cys2His2-type zinc fingers	5’-AGGCA	[25] [195]
CRE1	Cys2His2 type transcription factor	5’-SYGGRG	
*Neurospora crassa*			
**Positive regulators**			
**Name**	**Structure**	**Consensus region**	**References**
CLR-1/-2	Two zinc binuclear cluster	-	[145]
PacC	Three Cys2His2 zinc fingers	5’-GCCARG	[147]
*Neurospora crassa*			
**Positive regulators**			
**Name**	**Structure**	**Consensus region**	**References**
XLR1	Zinc binuclear cluster protein		[148]
NIT2	Single zinc finger protein	5’- TATCTA	[149]
**Negative regulators**			
**Name**	**Structure**	**Consensus region**	**References**
CRE1	Cys2His2 type transcription factor	5’-SYGGRG-	[152]
*Aspergillus spp.*			
**Positive regulators**			
**Name**	**Structure**	**Consensus region**	**References**
AmyR	Zn(II)2Cys6-binuclear cluster DNA-binding motif	5’-CGGN8CGG-3'	[194,-196]
AraR	Zn(2)Cys(6) binuclear cluster domain		[197,198]
PacC	Three Cys2His2 zinc fingers	5’- GCCARG	[22]
XlnR	Zinc binuclear cluster protein	5'-GGCTAAA	[48]
ClbR	Zn(II)2Cys6-binuclear cluster DNA-binding motif	CGG or CCG triplets	[174]
AreA	Highly conserved DNA binding motif comprising a Cys(4) zinc finger followed by a basic domain	5’-GATA (core sequence)	[182]
**Negative regulators**			
**Name**	**Structure**	**Consensus region**	**References**
CREA	Cys2His2 type transcription factor	5’-SYGGRG	[183-187]
CREB			
CREC			
